# A Population-Based Study on the Prevalence and Risk Factors of Chronic Kidney Disease in the Adult Population of Shiraz, Southern Iran

**DOI:** 10.31661/gmj.v0i0.935

**Published:** 2019-01-25

**Authors:** Marzieh Bakhshayeshkaram, Jamshid Roozbeh, Sayed Taghi Heydari, Behnam Honarvar, Mohammad Hossein Dabbaghmanesh, Maryam Ghoreyshi, Kamran Bagheri Lankarani

**Affiliations:** ^1^Health Policy Research Center, Institute of Health, Shiraz University of Medical Sciences, Shiraz, Iran; ^2^Shiraz Nephro-Urology Research Center, Shiraz University of Medical Sciences, Shiraz, Iran; ^3^Shiraz Endocrinology and Metabolism Research Center, Shiraz University of Medical Sciences, Shiraz, Iran

**Keywords:** Prevalence, Risk Factors, Chronic Kidney Disease

## Abstract

**Background:**

Currently, we are facing a significant increase in the new cases of the end-stage renal disease in developing countries. Hence, it seems vital to work on strategies aimed at reducing its development and progression. Determining the related risk factors can provide an insight into achieving these policymaking goals. Therefore, this study was conducted to identify risk factors associated with chronic kidney disease (CKD) in the Iranian adult population.

**Materials and Methods:**

This cross-sectional study was performed in Shiraz, Southern Iran, through a cluster random sampling technique that involved 819 subjects, including 340 male and 479 female adult participants. Factors such as the body mass index, waist circumference, blood pressure, and biochemical profile were determined. We evaluated the prevalence of CKD according to the glomerular filtration rate (GFR), as well as possible risk factors associated with it. GFR was calculated on the basis of the "Chronic Kidney Disease Epidemiology Collaboration" creatinine equation.

**Results:**

The cluster comprised 58.5% females and 41.5% males. The mean age of our participants was 43.0 ± 14.0 years. Our results showed that 16.6% of adult urban inhabitants in Iran had CKD (stages 3 to 5, eGFR ≤60), that is, GFR less than 60 mL/min/1.73 m2. The proportion of participants having hypertension, obesity, high waist circumference, diabetes mellitus, and history of cardiovascular disease was 17.3%, 19.3%, 35%, 9.4%, and 5.3%, respectively. Multiple regression analysis indicated an independent correlation between age, sex, dyslipidemia, and hypertension with CKD.

**Conclusion:**

This study indicates that CKD is a substantial health burden in Iranian adult population. Additionally, the results of this study addressed the importance of integrated strategies that aimed to identify, prevent, and treat noncommunicable diseases fueling the development of CKD.

## Introduction

Recent investigations have indicated a prominent increase in the incidence and prevalence of chronic kidney disease (CKD), especially in developing countries. Over the past three decades, CKD, also known as end-stage renal disease (ESRD), has emerged as a primary health concern, and it has shown to affect not only physical but also psychological (e.g., depression) and social conditions of patients (e.g., unemployment) [1]. It is important to have an early diagnosis and appropriate treatment to prevent further damage. In this regard, we need to have a reliable estimate of its epidemiology in developing countries. To the best of our knowledge, there is insufficient information in this regard [2]. The level of kidney function is best determined by a quantitative test named glomerular filtration rate (GFR). However, GFR itself is assessed through the renal clearance of a filtration marker. The most widely used marker by clinicians is serum creatinine [3]. Creatinine clearance has been the most common method of estimating the kidney function. However, the prevalence of CKD is calculated according to the GFR. Two of the most common GFR equations in adults are as follows: modification of diet in renal disease (MDRD) and Cockcroft-Gault (CG) [4]. These equations are used to classify end-stage CKD, monitor disease evolution, guide therapeutic measurements, and forecast prognosis. One of the suggested downsides of these two equations is the underestimation of GFR in individuals without severe renal condition [5]. A newer equation named “Chronic Kidney Disease Epidemiology Collaboration” (CKD-EPI) showed more reliable GFR estimates. It was more efficient in detecting preclinical end-organ changes than those calculated by other equations [6]. There are not sufficient studies about the incidence and prevalence of CKD in urban parts of Iran. In contrast to default expectations, results of a few available studies showed that its prevalence and incidence are more in developed countries than in developing countries [7]. To explore this disparity, more studies are needed. To the best of our knowledge, in studies on Iranian population, the CKD-EPI equation has not been applied to check GFR of the cluster sample. Hence, we decided to perform this population-based cross-sectional study in urban part of Iran on the basis of the CKD-EPI equation. We also checked for the most common associated risk factors, that is, hypertension (HTN), stroke, diabetes mellitus (DM), and cardiovascular disease (CVD). Also, we explored certain demographic and socioeconomic conditions, including age, gender, marital status, income, and educational level as the possible CKD risk factors.

## Materials and Methods

### Study Population

Between November 2011 and September 2012, this cross-sectional study was executed in Shiraz, Iran. Shiraz is the capital of Fars Province and a major metropolitan city in Southern Iran. We used proportion weight-based random cluster sampling according to the municipality zone, postal zip code, and home address. On the basis of the results of previous studies, to reach the objectives of our study, the sample size was calculated to be 800 subjects using the formula

$$N=\frac{\left(z_{1-\frac{\alpha }{2}}+z_{1-\beta })2p(1-p\right)}{d^{2}}$$

where d = 0:03, α = 0:05, z_1 – α/2_ = 1:96, z_1 – β_ = 0.80, and p = 0.116.

During this 10-month period, individuals were selected among all 7 Shiraz city zones. Anyone older than 18 years was invited via email to participate in the study based on the randomly selected addresses. Those who decided to participate in the study were assigned a day with the study team members. During the interview, detailed history and physical examination, as well as laboratory tests were collected and registered by an experienced team comprising physicians and nurses. The exclusion criteria included pregnancy, delivery in the last six months, acute kidney disease, and foreign nationals. Epidemiologists and clinical nephrologist collaborated to design standard questionnaires for this survey. These questionnaires were used to collect all information, including history taking, physical examinations, and demographics through a personal visit by medical team members. Evaluated demographics were age, sex, marital status, education, income, etc. Past medical history included DM, HTN, CVD, etc.

The institutional review board (IRB) and research ethics committee of Health Policy Research Center affiliated with Shiraz University of Medical Sciences, Shiraz, Iran, approved the study protocol. Before engaging in the study, informed consent was collected from each participant.

### Anthropometric Assessments

Two nurses, as part of the survey team, checked the anthropometrics. A standard scale was used to measure weight while the individual had worn light clothes. Through dividing weight (kg) by height (m^2^), the body mass index (BMI) was calculated. The setpoint for determining the waist circumference (WC) was the midpoint between the inferior border of the 12th rib and the iliac crest. Also, we considered the largest circumference of both hip sides as the hip circumference (HC). Through dividing WC (cm) by HC (cm), we calculated the waist-to-hip ratio. To record blood pressure (BP), we followed a unified protocol; BP was checked by a mercury sphygmomanometer in the right arm while the subject was sitting on a chair.

### Sample Collection and Biochemical Analysis

Subjects came to the clinic after an overnight 10-hour fasting, which was explained by team members over the phone or via email. Blood samples were collected to check fasting blood sugar (FBS), triglyceride (TG), total cholesterol (TC), low-density lipoprotein (LDL), and high-density lipoprotein (HDL). FBS was measured using the spectrophotometer. Serum TC, HDL-C, and TG concentrations were assessed by enzymatic reagents (Biosystems, Barcelona, Spain) with an A-25 Biosystems autoanalyzer. The Friedewald equation was used for estimating LDL concentrations indirectly from the measured levels of TG, HDL-C, and TC.

### Definition

BMI was categorized according to the World Health Organization (WHO) guidelines: 18.5 to 24.9 kg/m^2^ (normal), 25 to 29.9 kg/m^2^ (overweight), and ≥30 kg/m^2^ (obese). On the basis of WC measurements, the individuals were classified as abdominal obesity according to cutoffs recommended by the WHO, denoting more than 88 and higher than 102 of circumference for females and males, respectively. The normal waist-to-hip ratios were defined <0.85 in females and <0.9 in males. The CKD-EPI equation was used to estimate GFR [6]. In stages 1 and 2, GFR was ≥90 mL/min/1.73 m^2^ and 60 to 89 mL/min/1.73 m^2^, respectively. In stages 3, 4, and 5, GFR was 30 to 59 mL/min/1.73 m^2^, 15 to 29 mL/min/1.73 m^2^, and 15 mL/min/1.73 m^2^, respectively. The prevalence of CKD, defined as an estimated GFR<60 mL/min/1.73m^2^ and measured by CKD-EPI, was estimated as a whole, as well as by sex and the age group. The demographic and clinical variables were compared between the groups of patients with GFR less than 60 mL/min/1.73 m^2^ and individuals without CKD (GFR >90 mL/min/1.73 m^2^). HTN was defined as follows: (a) self-reported HTN (diagnosed by a physician or receiving medications for HTN); (b) systolic blood pressure ≥140 mm Hg; (c) average diastolic blood pressure ≥90 mm Hg; and (d) prehypertension characterized by the systolic blood pressure of ≥120 mm Hg and <140 mm Hg (with the diastolic pressure <90 mm Hg), or by a diastolic blood pressure of ≥80 and <90 mm Hg (with the systolic pressure <140 mm Hg). Individuals were classified as having dyslipidemia if they received lipid-lowering therapy or if they had at least one of the following conditions: the total cholesterol level ≥200, LDL-C level ≥130 mg/dL, triglyceride level ≥150 mg/dL, and low HDL-C level <40 mg/dL in males and low HDL-C level<50 mg/dL in females. Participants were classified as diabetics when they had either self-reported diabetes diagnosed by a physician and/or receiving medications for diabetes or fasting blood sugar ≥126 mg/dL. Individuals with a fasting glucose level of 100 to 125 mg/dL had impaired fasting glucose (IFG) or were considered as having prediabetes.

### Statistical Analysis

Statistical package for the social sciences (SPSS) version 22 (IBM Corporation, New York, USA) was used for statistical evaluation and analysis. Quantitative values were expressed as mean ± SD. We used univariate and multiple logistic regression tests to check any possible correlation between CKD and clinical factors. Besides, odds ratios (ORs) and 95% CIs were calculated. P-values less than 0.05 were considered statistically significant.

## Results

The study sample included 819 subjects. The mean age of the participants was 43.0 ± 14.0 years, ranging from 18 to 88 years. The cluster sample comprised 58.5% females and 41.5% males. Table-1 shows demographics, social data, and clinical information. Those with HTN, Pre-HTN, obesity, increased WC, increased WC/HC, and history of CVD were 17.3%, 13.5%, 19.3%, 35%, 53.6%, and 5.3%, respectively. Of the participants, 9.4% were diabetics, and 9.8% were prediabetics. The prevalence of dyslipidemia was 65.8%. The age-specific prevalence of GFR categories was estimated using the CKD-EPI equation (Table-2). The prevalence of GFR 60 to 89, 30 to 59, and <30 mL/min/1.73 m^2^ was 53.7%, 16%, and 1.1%, respectively. The prevalence of CKD defined as GFR<60 mL/min/1.73 m^2^ was 16.6%, including 14% in males and 19.4% in females. The prevalence of moderate to severe decrease in kidney function demonstrated by 60 mL/min/1.73 m^2^ was greater in females than in males (P = 0.028). The older age was strongly associated with the greater prevalence of mild, moderate, and severe decreases in kidney function (P<0.001). Using univariate analysis, CKD (GFR <60 mL/min/1.73 m^2^) was found to be significantly (P<0.05) associated with age, marital status, BMI, WC, diabetes, history of CVD, and HTN ( Table-3). In the multivariate logistic regression model, older participants, femaleness, HTN, and dyslipidemia were significantly associated with CKD ( Table-3).

## Discussion

The number of patients with CKD continues to increase worldwide. The incidence rates of ESRD have stabilized for many developed countries [8]. However, epidemiologic transition typified by a relative rise in incidence rates of ESRD is underway in many developing countries [9]. The burden of CKD is expanding quickly, as are the risk factors for kidney diseases such as DM, obesity, and HTN. The adverse consequences of CKD can be prevented or delayed through the early detection of these risk factors and early intervention strategies [10]. However, population-based, comprehensive studies relating to the prevalence of kidney damage in community are very limited in developing countries [11]. In this study, we showed that 16.6% of adult urban inhabitants in Iran had CKD (stages 3 to 5, eGFR ≤60). The prevalence of CKD varies among different regions and ethnic groups of the world. Several studies have documented the high prevalence of CKD in Iran. Recently, the prevalence of CKD reported in the northeast of Iran was 23.7% [12]. A recent meta-analysis also showed that the prevalence of CKD in Iran is markedly more than that reported in other developing countries and similar to developed countries [13]. Univariate analysis showed that risk factors such as age, BMI, WC, diabetes, HTN, dyslipidemia, and history of CVD are associated with CKD. But in the multivariate analysis, they were arbitrated to be insignificant, except in older female participants with HTN and dyslipidemia. Consistent with the results of other reports, our study showed a direct relationship between the prevalence of CKD and age [13]. Age is closely related to CKD because of the decrease in GFR associated with aging. On the other hand, certain factors have been shown to be associated with a greater risk of CKD, including smoking, dyslipidemia, HTN, DM, and obesity [14]. In this study, the prevalence of CKD in stages 3 to 5 was higher in females than in males, a finding that is in line with the results of a recent study reported from the United States. According to the System Annual Data Report, the prevalence of chronic renal failure was reported during the period between 2007 and 2012. The urinary albumin-to-creatinine ratio and a prevalence of decreased GFR were defined as an estimated GFR <60 mL/min/1.73 m^2^, which were more common among females than males [15]. However, this was a point of contention between the results of several studies [16-18]. An epidemiological study in France showed a higher incidence of CKD in males than females. This was consistent with the results of other cross-sectional studies reported from China [17, 18]. These discrepancies may be due to geographic and ethnic differences. HTN is a leading risk factor for ensuing decrease in GFR and CKD [19]. According to our results, univariate analysis showed a higher BMI and WC in individuals with end-stage CKD. High BMI and central obesity are extensively recognized risk factors for CKD [20]. Baseline BMI may identify patients at the increasing risk of progressive CKD [21]. However, associations illustrated between the prevalence of CKD and obesity evaluated by BMI are weak and inconsistent [22]. BMI indicates the composite assessment of lean mass, peripheral and abdominal adipose tissue, and bone mass rather than excess central fat, which is thought to aggravate the outcomes. WC appears to be a better marker of ESRD than BMI for obesity [22]. Contributing factors to the pathophysiology of CKD in obesity include the activation of the renin-angiotensin-aldosterone system, increased sympathetic nervous activity, insulin resistance, and oxidative stress [23]. Diabetes is another prominent risk factor for CKD because it is strongly associated with abdominal adiposity and WC [24]. Multivariate analysis revealed no significant association between CKD, higher BMI, WC, and diabetes, despite increasing values obtained by univariate analysis in individuals with CKD than those without CKD. These may be attributed to a close relationship between higher BMI, increasing WC, diabetes, and aging [25]. Our findings of univariate analysis showed an association between dyslipidemia and CKD. This association was also found by multivariate analysis. In this study, the prevalence of dyslipidemia was 65.8% in patients with stages 3 to 5 CKD. In progressive CKD, dyslipidemia often deteriorates the prevalence of dyslipidemia reported by 2001 to 2010 National Health and Nutrition Examination Survey, indicating an increasing trend from 45.5% in the CKD stage 1 to 67.8% in the CKD stage 4 [26]. CKD comprised the characteristic effects on major lipoprotein fractions with high triglyceride, low-HDL cholesterol concentrations, and altered lipoprotein composition [27]. The lipoprotein composition is also altered in CKD with increased small dense LDL and decreased larger LDL particles in CKD individuals [28]. Small, dense LDL particles are more atherogenic in CKD subjects than large LDL particles. The atherogenicity is also increased by the oxidation of small LDL particles [29]. These abnormalities in lipid metabolism supposedly play a possible role in progressive CKD [30]. This study had some limitations as well. First, GFR was not measured in a direct relationship with exogenous filtration markers. Second, the study did not measure albuminuria, which could underestimate the prevalence of CKD in our study.

## Conclusion

This study indicates that CKD is a substantial health burden in Iranian adult population. In multiple regression analysis, age, femaleness, dyslipidemia, and HTN were found to be associated with CKD. The results of our study underscore the importance of integrated strategies aimed at the prevention and curtailment of progressive noncommunicable diseases that ultimately fuel the development of CKD.

## Acknowledgment

This survey was funded by Shiraz University of Medical Sciences, Shiraz, Iran, and it was driven from the project number 104-12389. The authors would like to express their gratitude to Dr. Kabiri for linguistic editing of the manuscript.

## Conflict of Interest

None declared.

**Table 1:** Distribution of Demographic and Clinical Variables in Study Participants

	**Female**		**Male**		**Total**	
	**N (%)**	**Mean Age (SD)**	**N (%)**	**Mean Age (SD)**	**N (%) Total**	**Mean Age (SD)**
**Variables**	479 (58.5)	42.8 (14.5)	340 (41.5)	43.3 (13.3)	819 (100)	43.0 (14.0)
**Age (years)**						
<30	108 (22.6)	24.6 (2.6)	58 (17.1)	24.7 (2.6)	166 (20.3)	24.6 (2.6)
30-39	98 (20.5)	33.9 (2.7)	67 (19.7)	33.9 (3.0)	165 (20.2)	43.0 (14.0)
40-49	106 (22.2)	44.3 (3.0)	104 (30.6)	43.6 (2.5)	210 (25.7)	43.0 (14.0)
50-59	102 (21.3)	53.5 (2.8)	69 (20.3)	53.8 (2.8)	171 (20.9)	43.0 (14.0)
60-69	41 (8.6)	63 (2.9)	32 (9.4)	62.7 (2.7)	73 (8.9)	43.0 (14.0)
≤70	23 (4.8)	75.4 (4.9)	10 (2.9)	77.4 (1.8)	33 (4)	76.0 (5.2)
**Marital Status**						
Married	399 (83.5)	45.1 (13.8)	289 (85.0)	27.8 (9.0)	688 (84.1)	45.57 (13.12)
Single	79 (16.5)	30.9 (11.8)	51 (15)	46.1 (12.0)	130 (15.9)	29.70 (10.88)
**Education**						
Illiterate	29 (6.7)	43.3 (17.2)	19 (6.1)	42.5 (12.6)	48 (6.4)	43.0 (15.4)
Under Diploma	119 (27.4)	42.8 (14.4)	75 (20.4)	46.7 (13.6)	194 (25.9)	44.3 (14.2)
Diploma	142 (32.6)	42.6 (14.2)	119 (38)	42.7 (13.5)	261 (34.9)	42.5 (13.9)
University	145 (33.3)	44.4 (14.8)	100 (31.9)	41.9 (12.9)	245 (32.8)	43.3 (14.1)
**Body Mass Index (kg/m**^2^**)**						
<18.5	10 (2.1)	28.3 (16.7)	15 (4.5)	39.2 (17.1)	25 (3.1)	34.8 (17.4)
18.5-24.9	158 (33.3)	36.2 (14.4)	150 (45)	41.3 (13.3)	308 (38.2)	38.7 (14.1)
25-29.9	187 (39.5)	46.3 (13.2)	131 (39.3)	45.8 (13.2)	318 (39.4)	46.1 (13.2)
≤30	119 (25.1)	47.3 (12.7)	37 (11.1)	43.5 (11.3)	156 (19.3)	46.4 (12.4)
**Fasting Blood Sugar (mg/dL)**						
Normal	382 (79.7)	40.7 (14.2)	280 (82.4)	41.3 (12.6)	662 (80.6)	40.9 (13.6)
Prediabetes	47 (9.8)	48.1 (12.6)	33 (9.7)	48.8 (11.4)	80 (9.8)	48.4 (12.0)
Diabetes^a^	50 (10.4)	53.8 (12.1)	27 (7.9)	57.7 (11.1)	77 (9.4)	55.2 (11.8)
**Blood Pressure (mm Hg)**						
Normal	352 (73.5)	38.6 (12.4)	214 (63.3)	39.6 (10.7)	266 (69.3)	39(11.8)
Prehypertension	40 (8.4)	49.1 (13.8)	70 (20.7)	47.4 (14.7)	110 (13.5)	48.0 (14.4)
Hypertension^b^	87 (18.2)	56.6 (13.1)	54 (16.0)	53.1 (14.1)	141 (17.3)	55.3 (13.6)
**Lipid Profile (mg/dL)**						
Normal	122 (26.2)	38.9 (13.7)	148 (45.7)	41.6 (12.1)	270 (34.2)	40.4 (13.3)
Abnormal	344 (71.8)	44.1 (14.6)	176 (54.3)	44.8 (13.4)	520 (65.8)	44.4 (14.2)
Normal HDL	240 (51.5)	44.1 (14.7)	263 (79.9)	43.4 (13.0)	503 (63.3)	42.5 (13.2)
Abnormal HDL^c^	226 (48.5)	41.3 (14.3)	66 (20.1)	43.2 (14.0)	292 (36.7)	41.7 (14.2)
Normal LDL	347 (74.5)	40.6 (14.4)	267 (82.4)	42.6 (13.2)	614 (77.7)	41.5 (13.9)
Abnormal LDL^d^	119 (25.5)	48.8 (13.4)	57 (17.6)	46.7 (13.2)	176 (21.5)	48.8 (13.4)
Normal Cholesterol	279 (59.9)	38.9 (14)	226 (68.7)	41.9 (13.3)	505 (63.5)	40.3 (13.8)
Abnormal Cholesterol^e^	187 (40.1)	48.5 (13.4)	103 (31.3)	46.6 (12.4)	290 (36.5)	47.8 (13.1)
Normal TG	318 (69.1)	39.8 (14.1)	218 (67.3)	42.6 (13.3)	543 (68.3)	41 (13.8)
Abnormal TG^f^	142 (30.9)	49.3 (13.5)	106 (32.7)	45.0 (12.8)	252 (31.7)	47.4 (13.3)
**History of Cardiovascular disease**						
No	477 (94.5)	41.7 (13.8)	318 (94.9)	42.5 (12.8)	763 (94.7)	42.0 (13.4)
Yes	26 (5.5)	63.3 (11.5)	17 (5.1)	60.7 (8.1)	43 (5.3)	62.3 (10.3)

aaDiabetes: fasting blood sugar ≤126 mg/dL. bHypertension: Systolic ≤ 140 or diastolic ≤ 90 mm Hg. cAbnormal high density-lipoprotein (HDL) <40 for males and <50 for females. dAbnormal low-density lipoprotein (LDL) ≥130. eAbnormal cholesterol ≥200. fAbnormal triglyceride (TG) ≥150.

**Table 2:** Chronic Kidney Disease Epidemiology Collaboration (CKD-EPI) Equation-Based Glomerular Filtration Rate in Relation to Gender and Age

	**Mean Age (SD) in Years**	**Total No.**	**GFR** ^a^ ** ≤ 90**	**GFR 60-89**	**GFR** **30-59**	**GFR 15-29**	**GFR < 15**
**All Participants**	43.0 (14.3)	819	232 (29.2)	426 (53.7)	127 (15.5)	5 (0.6)	4 (0.5)
**Females**	42.8 (14.5)	479	133 (28.7)	241 (51.9)	84 (18.1)	4 (0.9)	2 (0.4)
**Males**	43.3 (13.3)	340	99 (30.1)	184 (55.9)	43 (13.1)	1 (0.3)	2 (0.6)
**Age (years)**							
<30	24.6 (2.6)	166	78 (49.2)	74 (46.5)	5 (3.1)	1 (0.6)	1 (0.6)
30-39	43.0 (14.0)	165	65 (40.6)	84 (52.5)	10 (6.3)	1 (0.6)	0 (0.0)
40-49	43.0 (14.0)	210	54 (26.0)	120 (57.7)	32 (15.4)	1 (0.5)	1 (0.5)
50-59	43.0 (14.0)	171	28 (17.2)	102 (62.6)	30 (18.4)	1 (0.6)	2 (1.2)
60-69	43.0 (14.0)	73	7 (10)	38 (54.3)	25 (35.7)	0 (0.0)	0 (0.0)
≤70	76.0 (5.2)	33	0 (0.0)	7 (21.2)	25 (75.8)	1 (3.0)	0 (0.0)

**GFR:** Glomerular filtration rate. aGFR (mL/min/1.73 m2) based on CKD-EPI equation (number/percentage).

**Table 3:** Univariate and Multivariate Analyses c Risk Factors for Chronic Kidney Disease in Studied Population

**Variable**	**GFR ≤ 90 (%), n=232**	**CKD Stages ≤60 (%), n=136**	**P** ***-*** **Value** ^a^	**OR** ^b^ ** (95% CI)**	**OR** ^c^ ** (95% CI)**	**P-Value** ^a^
**Age (years)**						
18-34	120 (90.2)	13 (9.8)	<0.001	1	1	<0.001^a^
35-49	77 (66.4)	39 (33.6)		4.68 (2.35-9.32)	5.22 (2.30-11.84)	
>50	4 (29.4)	84 (70.6)		22.15 (11.06-44.39)	31.06 (12.67-76.16)	
**Sex**						
Male	99 (68.3)	46 (31.7)	0.094	1	1	0.012^a^
Female	133 (59.6)	90 (40.4)		1.46 (0.94-2.26)	2.08 (1.17-3.70)	
**Marital Status**						
Single	54 (80.6)	13 (19.4)	0.001	1	1	0.795
Married	178 (59.1)	123 (40.9)		2.87 (1.50-5.49)	.84 (0.34-2.09)	
**Education**						
Illiterate	14 (6.8)	9 (6.8)	0.698	1	¼	¼
Under Diploma	57 (24.8)	41 (30.1)		1.22 (0.48-3.11)		
Diploma	81 (35.0)	41 (30.1)		0.86 (0.34-2.17)		
University	78 (33.5)	45 (33.1)		0.99 (0.40-2.49)		
**Body Mass Index**						
18.5-24.9	116 (49.8)	41 (30.1)	0.001	1	1	0.913
≤30	33 (14.3)	33 (24.1)		2.79 (1.52-5.10)	0.95 (0.53-1.70)	
**Waist Circumference**						
Normal	174 (75.0)	66 (48.5)	<0.001	1	1	0.054
High	58 (25.0)	70 (51.5)		3.16 (2.00-5.00)	1.91 (0.98-3.72)	
**Hypertension**						
No	179 (77.5)	76 (55.9)	<0.001	1	1	0.030^a^
Hypertension	25 (10.8)	43 (31.6)		4.05 (2.31-7.10)	2.16 (1.07-4.35)	
**Diabetes**						
No	197 (84.9)	97 (71.3)	0.021	1	1	0.136
Diabetes	19 (8.2)	22 (16.2)		2.35 (1.21-4.55)	0.53 (0.23-1.21)	
**Dyslipidemia**						
No	89 (38.5)	33 (23.9)	0.004	1	1	0.048^a^
Yes	143 (61.5)	103 (76.1)		2.00 (1.24-3.22)	1.84 (1.00-3.38)	
**History of Cardiovascular Disease**						
No	223(65.2)	119 (34.8)	<0.001	1	1	0.658
Yes	6 (28.6)	15 (71.4)		4.68 (1.77-12.39)	0.78 (0.25-2.36)	

**GFR:** glomerular filtration rate; CKD: chronic kidney disease. ^a^ p value<0.05 ^b^Odds ratio (OR) and 95% CI. ^c^Multiple logistic regression model analysis; Adjusted OR and 95% CI.
